# Bibliographical Notices

**Published:** 1855-01

**Authors:** 


### EDITORIAL DEPARTMENT.

### BIBLIOGRAPHICAL NOTICES.

*The Principal Forms of the Skeleton and of the Teeth.* By Prof. R. Owen, F. R. S., &c. Philadelphia, Blanchard & Lea, 1854.

Transcendental anatomy is a subject comparatively new to the scientific world. Oken and Gœthe, we understand, both have a claim to first detecting the vertebral character of the cranium, from which discovery arose the whole system of homological anatomy.

This school of anatomists deals with homologies, that is, with the science of corresponding parts, and endeavors by numerous comparisons and careful study, to deduce some formula to which the various modifications of these parts can be referred. Some of them are so elated by the new ideas thus developed that they undertake to sneer at the old teleogical school, which studied the same modifications with reference to their adaptation to external nature.

The work before us combines both methods, and, therefore, elicits more truth. It is a masterly review of the great fundamental forms of the vertebrate skeleton and of the comparative anatomy of the teeth.

*A Manual of Pathological Anatomy.* By C. Handfield Jones, M. B., F. R. S., &c., and Edward H. Sieveking, M. D. First American edition, revised, with 397 illustrations. Philadelphia, Blanchard & Lea, 1854.

The authors of this book are well known as assiduous students of pathology and pathological anatomy. They have had abundant opportunities for prosecuting their favorite study, and come before the public with no small claims upon attention.

The work is a summary of the principles of pathological anatomy. It begins very properly with some general observations on the science, and gives an account of the changes in the blood, which lay the foundation of so many morbid alterations. It then passes on to consider the various textural changes which may occur in any tissue. From this the transition is easy to tumors. This part is closed by an account of parasites. Then follows in regular order, the pathological anatomy of the nervous system, of the organs of circulation and respiration, of the alimentary canal, of the urinary apparatus, of the organs of generation, of the joints and of the osseous system.

The writers have not only distributed their labor, but they have published that distribution to the public. Each article has appended to it the name of its author.

The American edition has been increased by many additional cuts, not to be found in the London edition. The illustrations of urinary deposits are very numerous.

---

*Physicians' Visiting List for* 1854. Philadelphia, Lindsay & Blakiston.

THIS book has been long enough known to the medical profession to make itself felt as a necessity. It is certainly the most commodious method of keeping an account of a physician's daily business, of which we have any knowledge.

---

*American Journal of Science and Arts for November.*

CONTENTS: Bache on Tides at Key West; Dana on Geographical Distribution of Crustacea; Hunt on Map Projections; Forbes on the Educational Uses of Museums; De la Rive on the cause of the Aurora Borealis; Shepard on Meteoric Iron from Senora; J. L. Smith, re-examination of American Minerals; Correspondence of M. Jerome Nickles; Cornell on the Nomenclature of the Metals contained in Columbite and Tantalite; Murchison's Siluria; Brush on the Chemical Composition of Clintonite; Genth, Contributions to Mineralogy; Scientific Intelligence.

We have repeatedly taken occasion to call the attention of our readers to this admirable journal, and now, at the commencement of the year, renew our recommendation to those who take an interest in science, to subscribe to it.

---

*On the Construction, Organization and General Arrangements of Hospitals for the Insane.* By THOMAS S. KIRKBRIDE, M. D. Philadelphia, Lindsay & Blakiston.

THE author of this work is the physician to the Pennsylvania Hospital for the Insane, and consequently speaks with authority. The book is a very comprehensive treatise on all that pertains to the comfort and the proper treatment of the unfortunate class of patients over whom its author has been accustomed to preside.

*A Treatise on Dentistry.* By Samuel Fowell. London, p. 80.

The author's object in writing this small treatise has been to furnish the public with sound practical information upon the teeth—in this he has succeeded, for although written in a popular form, it embraces much scientific information. R.

*Facts for the People, Relating to the Teeth*—Showing their influence upon the health, speech and looks, with directions for their care and preservation. By T. D. Thompson, D. D. S., of the Baltimore College. Boston, B. B. Mussey and Company, pp. 251, 12mo., 1854.

The above is the title of a popular treatise on the teeth. The subjects embraced in it are those in which all are more or less interested, being connected with the comfort and health of almost every individual. They are very judiciously arranged and treated in a clear and satisfactory manner, and while the book professes to be little more than a compilation, it is one that should be in every family. It contains much useful information and the non-professional reader can scarcely fail to derive advantage from its perusal. We hope it may have an extensive circulation.

